# Bilingual Cortical Control of Between- and Within-Language Competition

**DOI:** 10.1038/s41598-017-12116-w

**Published:** 2017-09-18

**Authors:** Viorica Marian, James Bartolotti, Sirada Rochanavibhata, Kailyn Bradley, Arturo E. Hernandez

**Affiliations:** 1 Northwestern University, Evanston, IL 60208 USA; 2 University of Houston, Houston, TX 77204 USA; 3 Icahn School of Medicine at Mount Sinai, New York, NY 10029 USA

## Abstract

The human capacity to master multiple languages is remarkable and leads to structural and functional changes in the brain. Understanding how the brain accommodates multiple languages simultaneously is crucial to developing a complete picture of our species’ linguistic capabilities. To examine the neural mechanisms involved in processing two languages, we looked at cortical activation in Spanish-English bilinguals in response to phonological competition either *between* two languages or *within* a language. Participants recognized spoken words in a visual world task while their brains were scanned using functional magnetic resonance imaging (fMRI). Results revealed that *between*-language competition recruited a larger network of frontal control and basal ganglia regions than *within*-language competition. Bilinguals also recruited more neural resources to manage between-language competition from the dominant language compared to competition from the less dominant language. Additionally, bilinguals’ activation of the basal ganglia was inversely correlated with their executive function ability, suggesting that bilinguals compensated for lower levels of cognitive control by recruiting a broader neural network to manage more difficult tasks. These results provide evidence for differences in neural responses to linguistic competition *between* versus *within* languages, and demonstrate the brain’s remarkable plasticity, where language experience can change neural processing.

## Introduction

One of the most groundbreaking and exciting findings in neuroscience in recent decades is the extent to which experience changes the brain. Whether it is due to musical training^1^, spatial navigation^2^, or motor skill development^3^, the brain remains highly plastic throughout life. Of all human experiences, language use has arguably the greatest potential to generate widespread neural changes, given its fundamental role in human function and the broad neural network essential to language processing. One example of a linguistically and cognitively demanding experience that causes substantial structural^4,5^ and functional^4,6^ changes in the brain is bilingualism. Bilinguals’ ability to switch between languages and maintain separation between them may appear effortless, but this seamlessness masks a complex interactive cognitive architecture. The aim of the current investigation is to examine the neural mechanisms enabling bilinguals to control their two languages during spoken language comprehension.

The need to maintain two languages provides unique challenges for bilinguals and affects neural processing of language and executive function. For example, behavioral and neuroimaging studies have shown that when bilinguals perform a language comprehension task in one language, the other non-target language also becomes activated^7–9^. Because bilinguals’ two languages are consistently coactivated, bilinguals’ brains adapt to manage language access differently than monolinguals. This experience managing linguistic activation appears to also extend to non-linguistic tasks. For example, bilinguals’ use of executive function in non-linguistic attentional control tasks has revealed differences compared to monolinguals in the recruitment of structures such as the prefrontal cortex and the anterior cingulate cortex^10–12^.

Activation of non-target words during language comprehension is not itself unique to bilinguals. As listeners hear speech input unfold over time, similar-sounding words become activated^13^. These lexical items compete for selection^14,15^, and must be inhibited before language comprehension can proceed. Both monolingual^14,15^ and bilingual listeners^16,17^ experience lexical competition during spoken language comprehension. Compared to monolinguals who face phonological competition only within a single language, bilinguals experience competition both within one language as well as between their two languages. For example, when Russian-English bilinguals are instructed (in one language only) to find an item on a visual display (e.g., “Click on the *shovel*”), they make eye movements to objects that are phonologically similar both in English (*shark*) and in Russian (*sharik*, Russian for “balloon”)^17^. Bilinguals’ fixations to both between- and within-language competitor objects, even when only one language is being used, suggest that lexical items in both languages are activated simultaneously.

Due to the highly interactive nature of a bilinguals’ two languages, cognitive control mechanisms are required to overcome competition. This suggests that bilinguals’ management of linguistic competition may be associated with their general executive control abilities^12^. Indeed, bilinguals have been shown to use general executive control to manage switching between their two languages in production^18–20^ and comprehension^21^. The link between executive control resources and the management of phonological competition within a single language has also been investigated and revealed that bilinguals recruit an efficient network of control regions to overcome within-language competition^22^. This efficient deployment of neural resources observed in bilinguals provides additional empirical support for the recently-proposed Bilingual Anterior to Posterior and Subcortical Shift model (BAPSS)^4^. The BAPSS model proposes that, with increased second language experience, bilinguals shift their processing load during executive tasks from frontal control areas, like the dorsolateral prefrontal cortex and anterior cingulate, to posterior perceptual areas and subcortical regions, such as the basal ganglia.

The present research aims to use phonological competition resolution to look at cortical processing of between- and within-language competition. The neural underpinnings of phonological competition within a single language have been shown in both monolingual and bilingual speakers. For example, English monolingual speakers activate frontal and temporal language regions in response to phonological competition^23^, particularly the left supramarginal gyrus and the left inferior frontal gyrus. For bilinguals, the neural correlates of overcoming competition arising within a single language have been elucidated^22^, but the cortical regions subserving between-language competition remain unknown. In response to within-language competition, bilinguals’ brains efficiently recruit frontal executive control regions, as they are able to manage competition without exhausting additional cortical resources relative to when competition is not present. Phonological competition that occurs between two languages, however, may present additional challenges for the bilingual, because of the additional need to control activation of the non-target language. In the current paper, we seek to compare how phonological competition that emerges between two languages and competition arising within a single language are managed in bilinguals.

The two main objectives of the present study are: (1) To compare the neural resources required to overcome between-language versus within-language competition; and (2) To examine the relationship between language coactivation and cognitive control. We predict that when confronted with between-language competition, bilingual listeners will recruit a broader network of frontal-control regions than that required by within-language competition. Because of the link between linguistic and non-linguistic cognitive control, we expect that individual differences in inhibitory control skill will be related to degree of neural activation in response to phonological competition.

## Results

To examine the neural mechanisms involved in processing two languages, we compared cortical activation in Spanish-English bilinguals in response to between- versus within-language phonological competition. Participants completed a spoken word recognition task using the visual world paradigm while in a functional magnetic resonance imaging (fMRI) scanner, under four experimental conditions: English within-language (English target, English competitor), English between-language (English target, Spanish competitor), Spanish within-language (Spanish target, Spanish competitor), and Spanish between-language (Spanish target, English competitor). Neural responses to phonological competitors were compared to baseline trials where no phonological competitors were present. In addition, participants completed assessments of phonological working memory, inhibitory control, and language proficiency outside the scanner.

### Accuracy and Response Time

For both accuracy and RT, the effects of Competition (competitor, unrelated), Language (target in English or in Spanish), and Type (between-language, within-language) and their interactions were analyzed using logistic (for accuracy) or linear (for RT) mixed effect regression (using the lme4 package^24^ in the R statistical computing environment^25^), including subject and item as random effects.

Accuracy was high overall (*M* = 91.57%, *SD* = 27.79) and there were no significant main effects, but there was a significant Language by Type interaction (*Estimate* = 19.4, *SE* = 9.0, *z* = 2.161, *p* < 0.05). Follow-up pairwise comparisons did not reveal any significant differences between the four levels of Language and Type (*p*s > 0.1). To account for a crossover interaction of Language and Type, we ran a new model including fixed effects of Competition, Target-Language, and Competitor-Language. This follow-up model was used to determine whether the language of the competitor, rather than its relation to the target (i.e., within- or between-languages) affected accuracy. We found a significant main effect of Competitor-Language (*Estimate* = 9.7, *SE* = 4.5, *z* = 2.161, *p* < 0.05), with lower accuracy for the English-Competitor condition, 86.67%, *SE* = 0.032, compared to the Spanish-Competitor condition 96.35%, *SE* = 0.032. These results indicate that as a competing language, English, which was the participants’ dominant language, affected task performance more than Spanish did. Outlier RTs (greater than the global mean plus two standard deviations) were replaced with M + 2.5 SDs (2.80% of trials). RTs on correct trials were 1785.18ms (*SD* = 383.35) overall. No significant main effects or interactions were observed (the same pattern of results was observed in an analysis removing outlier RTs entirely, with no significant main effects or interactions).

### Functional Neuroimaging

#### Comparing between-language versus within-language competition

**Figure 1:**
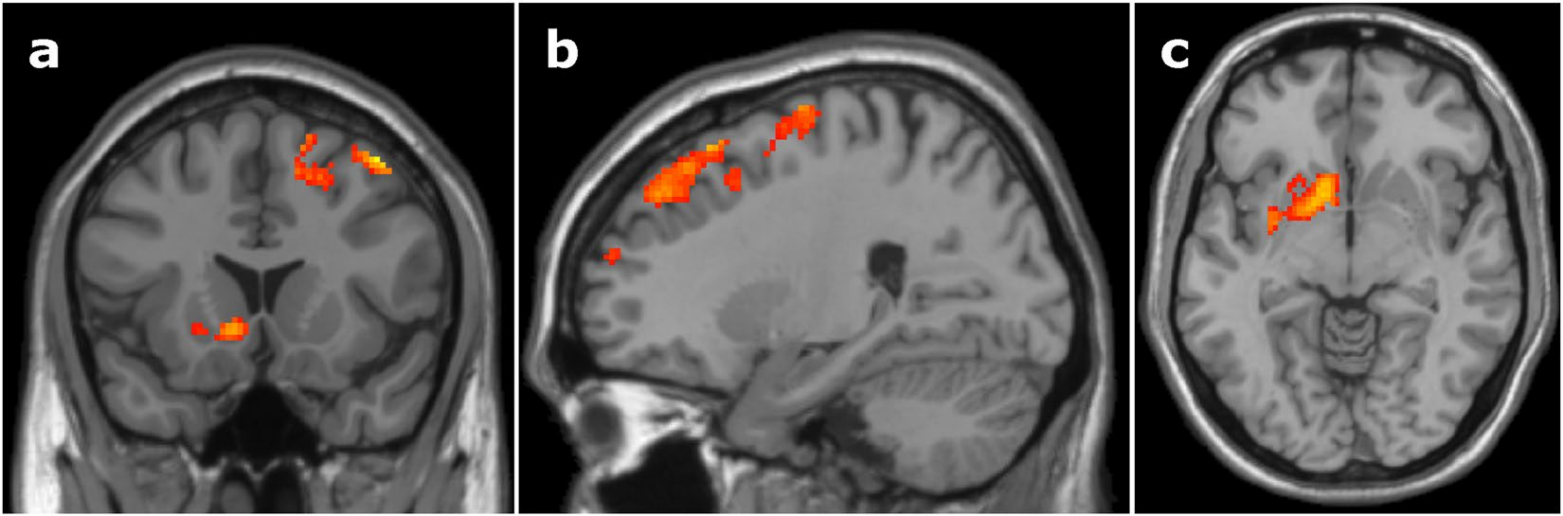
Between-language versus within-language phonological competition. Bilinguals showed increased activation in the left putamen and caudate, as well as the right middle frontal gyrus and superior frontal gyrus during between-language competition compared to within-language competition. (**a**) Coronal slice at y = 16, (**b**) sagittal slice at x = 24, (**c**) axial slice at z = −6, MNI template. Cluster-level significance of *p* < 0.05 based on Monte Carlo simulations with a voxel-level threshold of *p* < 0.025 and minimum cluster size of 509 contiguous voxels.

To examine differences in the cortical resources recruited to manage between-language versus within-language phonological competition across languages, we ran a 2 (Condition: between-language, within-language) by 2 (Language: English, Spanish) within-subject ANOVA on the competitor > unrelated-filler contrasts. There was a main effect of Condition; bilinguals recruited more cortical resources to manage between-language competition, where there was increased activation of the left putamen and caudate, as well as the right middle frontal gyrus and superior frontal gyrus (Fig. 1, Table 1C).

**Table 1:** Effects of phonological competition between- and within-language.

Cortical Region	Cluster Size	MNI Coordinates		
		x	y	z
**(** ***A*** **)** ***Between-language competition*** > ***Unrelated***				
Competition > Unrelated				
Left middle frontal gyrus	534	−36	18	52
Left superior frontal gyrus		−22	18	60
Right middle frontal gyrus	1869	34	4	52
Right superior frontal gyrus		22	40	44
Left caudate	634	−10	12	−4
Left putamen		−26	8	12
Right caudate	689	8	18	8
Right putamen		36	0	−2
Unrelated > Competition				
No suprathreshold clusters	—	—	—	—
**(** ***B*** **)** ***Within-language competition*** > ***Unrelated***				
Competition > Unrelated				
No suprathreshold clusters	—	—	—	—
Unrelated > Competition				
No suprathreshold clusters	—	—	—	—
**(** ***C*** **)** ***Between-language*** > ***Within-language competition***				
Between-language > Within-language competition				
Right middle frontal gyrus	1244	42	14	54
Right superior frontal gyrus		20	24	58
Left putamen	616	−12	10	−6
Left caudate		−6	2	12

*Note*: Voxels thresholded at *p* < 0.025 with a minimum cluster size of k = 509 contiguous voxels to yield a cluster threshold of *p* < 0.05. Coordinates indicate maximum intensity voxel peaks within a cluster.

#### Between-language competition

**Figure 2:**
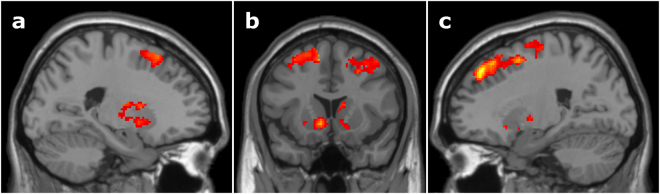
Between-language phonological competition versus matched unrelated trials. Bilinguals showed increased activation in bilateral middle frontal gyrus and superior frontal gyrus, as well as bilateral caudate and putamen, during between-language competitor trials compared to trials with no competitors present. (**a**) Sagittal slice at x = −22, (**b**) coronal slice at y = 14, and (**c**) sagittal slice at x = 22, MNI template. Cluster-level significance of *p* < 0.05 based on Monte Carlo simulations with a voxel-level threshold of *p* < 0.025 and minimum cluster size of 509 contiguous voxels.

Overall effects of between-language competition were assessed using a one-way ANOVA on the contrast comparing between-language competitors and their matched unrelated trials. During between-language competitor trials compared to the no-competition filler trials (between-language competitor > unrelated contrasts), bilinguals showed increased bilateral activation of the middle frontal gyrus and superior frontal gyrus and increased activation of bilateral caudate and putamen (Fig. 2, Table 1A). Comparisons of unrelated > between-language competitor trials showed no increased activation in any regions examined.

#### Within-language competition

Overall effects of within-language competition were assessed with a one-way ANOVA on the contrast comparing within-language competitors and their matched unrelated trials. No significant clusters were identified for either the within-language competitor > unrelated or the unrelated > within-language competitor contrasts in any brain regions examined (Table 1B).

#### Language-specific effects during between-language competition

**Figure 3:**
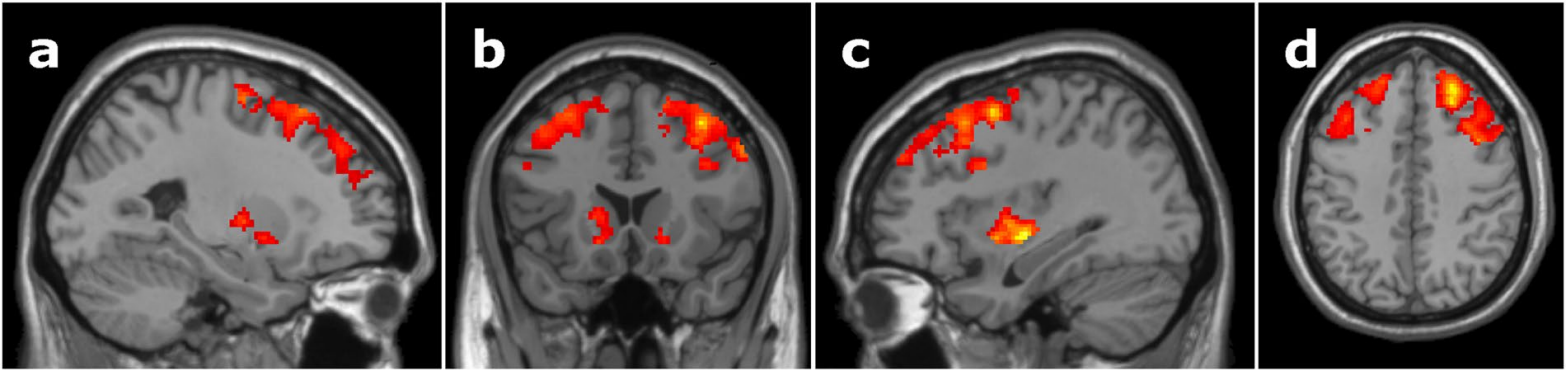
Spanish-English between-language phonological competition versus matched unrelated trials. Compared to trials during which there were no competitors, when receiving language input in Spanish and facing competition from English, bilinguals showed increased bilateral activation of the caudate and putamen, as well as bilateral inferior frontal gyrus, middle frontal gyrus, and superior frontal gyrus. (**a**) Sagittal slice at x = −26, (**b**) coronal slice at y = 18, (**c**) sagittal slice at x = 34, (**d**) axial slice at z = 42, MNI template. Cluster-level significance of *p* < 0.05 based on Monte Carlo simulations with a voxel-level threshold of *p* < 0.025 and minimum cluster size of 509 contiguous voxels.

Planned comparisons on the effect of between-language competition in each language were run using one-way ANOVAs comparing between-language competitors to matched unrelated trials, separately for each language. When auditory cues were received in Spanish, between-language competitor trials resulted in greater activation of bilateral inferior frontal gyrus, middle frontal gyrus, superior frontal gyrus, as well as the caudate and putamen bilaterally, compared to the no-competitor filler trials (Fig. 3, Table 2A). When participants heard the auditory target cue in English, no significant clusters were identified (Table 2B).

**Table 2:** Language-specific effects of between-language phonological competition.

Cortical Region	Cluster Size	MNI Coordinates		
		x	y	z
**(** ***A*** **)** ***Spanish-English between-language competition***				
Competition > Unrelated				
Left superior frontal gyrus	1652	−18	44	42
Left middle frontal gyrus		−30	18	54
Left inferior frontal gyrus		−36	34	26
Right superior frontal gyrus	2707	22	38	44
Right middle frontal gyrus		34	18	50
Right inferior frontal gyrus		54	24	34
Left putamen	949	−22	4	−6
Left caudate		−10	6	−10
Right putamen	763	32	−12	−4
Right caudate		16	18	−6
Unrelated > Competition				
No suprathreshold clusters	—	—	—	—
**(** ***B*** **)** ***English-Spanish between-language competition***				
Competition > Unrelated				
No suprathreshold clusters	—	—	—	—
Unrelated > Competition				
No suprathreshold clusters	—	—	—	—
**(** ***C*** **)** ***Spanish-English versus English-Spanish between-language competition***				
Spanish-English > English-Spanish competition				
Right superior frontal gyrus	618	30	28	54
Right middle frontal gyrus		40	24	40
Right inferior frontal gyrus		52	28	22
English-Spanish > Spanish-English competition				
No suprathreshold clusters	—	—	—	—

*Note*: Voxels thresholded at *p* < 0.025 with a minimum cluster size of k = 509 contiguous voxels to yield a cluster threshold of *p* < 0.05. Coordinates indicate maximum intensity voxel peaks within a cluster.

**Figure 4:**
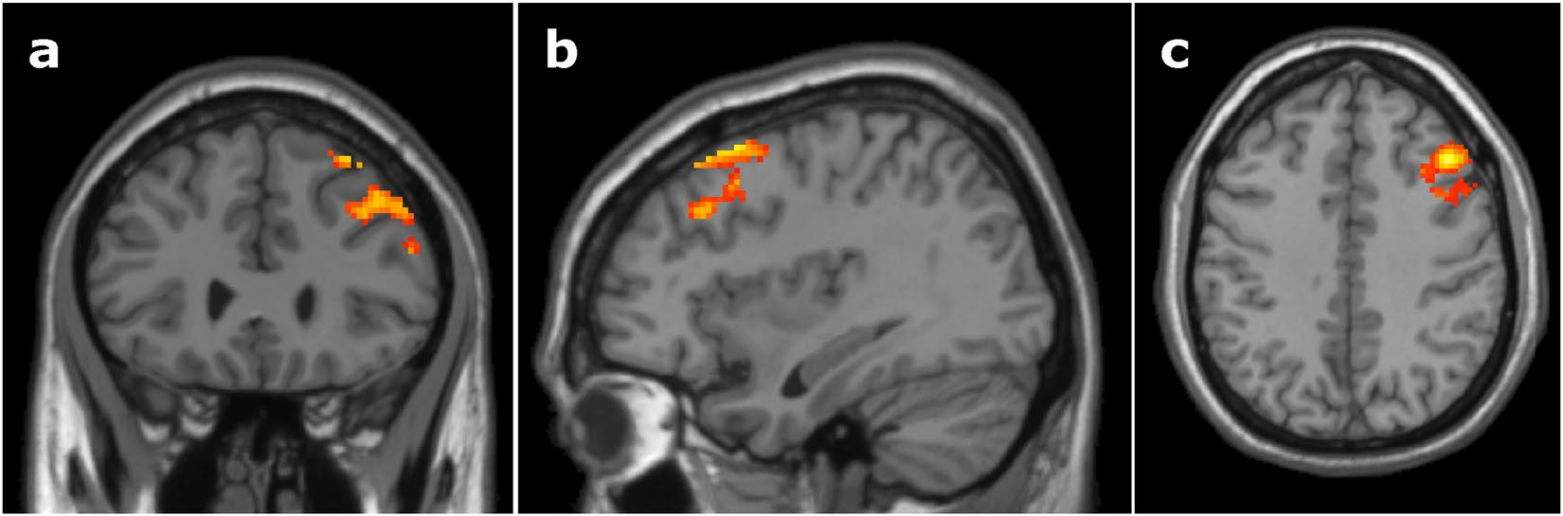
Language-specific activation during phonological competition. Bilinguals showed increased activation of the right middle frontal gyrus, superior frontal gyrus, and inferior frontal gyrus during trials where the language input was in Spanish and the between-language competitor was from English, compared to trials where the language input was in English and the between-language competitor was from Spanish. (**a**) Coronal slice at y = 28, (**b**) sagittal slice at x = 36, (**c**) axial slice at z = 40, MNI template. Cluster-level significance of *p* < 0.05 based on Monte Carlo simulations with a voxel-level threshold of *p* < 0.025 and minimum cluster size of 509 contiguous voxels.

To further analyze language-specific effects of between-language competition, we ran an additional ANOVA comparing the competitor > unrelated-filler contrasts in each language (Spanish-English between-language competition versus English-Spanish between-language competition). When listening to auditory instructions in Spanish and facing between-language competition from English, bilinguals showed increased activation of the right middle frontal gyrus, superior frontal gyrus, and inferior frontal gyrus, compared to when receiving auditory input in English and facing between-language competition from their native language, Spanish (Fig. 4, Table 2C). The English-Spanish between-language competition > Spanish-English between-language competition contrasts did not yield any significant clusters.

#### Correlations with proficiency and inhibitory control

To examine the relationship between linguistic and non-linguistic inhibitory control, we correlated bilinguals’ Simon inhibition scores and their overall RTs on the Simon task with individual effect sizes in task-activated ROIs for each of the four competitor contrasts (English within-language, Spanish within-language, English-Spanish between-language, and Spanish-English between-language). We also examined the relationship between non-dominant language proficiency and phonological competition by correlating bilinguals’ self-reported proficiency with effect sizes in task-activated ROIs.

Four ROIs were identified for overall between-language competition (Fig. 2), including right middle frontal gyrus (MFG)/superior frontal gyrus (SFG), left MFG/SFG, right putamen, and left putamen. We found that bilinguals with better (smaller) Simon inhibition scores had decreased activation in the right putamen in response to receiving input in Spanish and facing competition from English, *R* ^2^ = 0.41, *p* < 0.05. We also found a marginal correlation between Simon inhibition and activation in the right MFG/SFG, *R* ^2^ = 0.21, *p* < 0.1, where better Simon inhibition was associated with decreased activation of right MFG/SFG in response to receiving input in English and facing competition from Spanish. Further, we found that bilinguals with faster overall Simon RTs displayed decreased left MFG/SFG activation when receiving input in English and facing competition from Spanish, *R* ^2^ = 0.62, *p* < 0.05. There were no correlations between Simon task performance and activation in the left caudate/putamen, and no correlations between proficiency and neural activation.

Two ROIs were identified from the between-language versus within-language competition effect (Fig. 1), including left putamen/caudate and right MFG/SFG. We found a marginal correlation in the right MFG/SFG, *R* ^2^ = 0.28, *p* < 0.1, where better Simon inhibition was associated with decreased activation in response to English input and Spanish competition. No correlations were found with the left putamen/caudate.

Four ROIs were identified from the Spanish-English between-language competitor versus unrelated analysis (Fig. 3), including right MFG/SFG, left MFG/SFG, right putamen/caudate, and left putamen/caudate. When receiving language input in Spanish and facing English competitors, bilinguals with better (smaller) Simon inhibition scores had decreased activation in the right putamen in response to receiving input in Spanish and facing competition from English, *R* ^2^ = 0.24, *p* < 0.1. There were no correlations with Simon inhibition or overall RTs detected in right or left MFG/SFG or in the left putamen/caudate. We found correlations between subcortical activation and proficiency, where greater non-dominant language proficiency was associated with increased activation when receiving input in Spanish and facing competition from English in both left putamen, *R* ^2^ = 0.50, *p* < 0.01, and right putamen, *R* ^2^ = 0.25, *p* < 0.1. One ROI was identified from the Spanish-English between-language competition versus English-Spanish between-language competition analysis (Fig. 4) in right MFG/SFG, but no correlations were found between cortical activation and Simon inhibition or Simon task RTs.

## Discussion

Bilinguals’ ability to seamlessly switch between two distinct communication systems masks the considerable control exerted at the neural level. Spoken language comprehension is especially taxing, because the bilingual listener does not control the language of the input, and spoken words unfold gradually over time. To cope with this uncertainty in the input, bilinguals start by activating words in both languages that partially overlap with the auditory signal before selecting the best match. The current study examined the neural networks involved in processing two languages, offering the first comparison of how bilinguals control phonological competition that arises between both of their languages or within one language.

We found that the size and type of the neural network that bilinguals recruited to resolve phonological competition differed depending on the source of competition. When competition occurred between two languages, bilinguals recruited additional frontal control and subcortical regions, specifically the right middle frontal gyrus, superior frontal gyrus, caudate, and putamen, compared to competition that occurred within a single language. This difference in recruitment of brain regions suggests that between-language competition may require additional effort to suppress activation of the non-target language. Increased putamen and caudate activity in the between-language condition relative to the within-language condition is also in line with research suggesting that these areas are involved in cognitive and motor control^26–29^. The neural patterns observed in the present experiment provide additional empirical support for the BAPSS model^4^ and are consistent with the model’s proposal that between-language competition is initially managed by an extensive network of frontal regions, but later relies less on the frontal network and more on subcortical structures, such as the putamen and caudate. Even within our group of highly proficient bilinguals, the activation of subcortical structures was found to be related to small differences in proficiency. Specifically, as proficiency in the non-dominant language increased, so too did activation in the putamen in response to competition that occurred between languages.

We also found that the neural mechanisms recruited by bilinguals to resolve phonological competition differed depending on the language of competition. Specifically, relative proficiency in the two languages made a greater difference than age of acquisition in determining neural activation. The language with higher proficiency, English, showed more extensive activity than participants’ native language, Spanish. This is consistent with previous literature on bilingual language processing, showing that proficiency is more important than age of acquisition in determining cortical activation^30,31^. Although counterintuitive, earlier age of acquisition does not always lead to higher proficiency^32^, as is the case for the participants in the current study, who were Spanish native and English dominant, due to growing up in a country where English was the majority language, including in all academic settings. During between-language competition, when bilinguals received auditory input in Spanish and faced competition from English (the more dominant language), additional frontal control regions were recruited, including the right middle frontal gyrus and superior frontal gyrus, compared to when they received input in English and faced competition from Spanish (the less dominant language). This neural pattern suggests that additional cognitive resources are required to suppress competition from the more dominant language than competition from the less dominant language. These results are in line with cognitive models such as the Revised Hierarchical Model^33^, which propose that bilinguals access words differently as their second language proficiency increases; specifically, access to the second language may become more automatic with increasing proficiency, rather than controlled. Just as bilinguals with higher second language proficiencies have been shown to have decreased prefrontal activation relative to low-proficiency bilinguals^34^, bilinguals in the current study also showed less activation of frontal control regions when processing target words in English, which, although acquired second, was their dominant and more automatic and proficient language. Thus, proficiency in a language may be more important than age of acquisition for predicting the degree of competition experienced across languages.

Notably, these neural differences in response to phonological competition were observed despite minimal behavioral effects (i.e., accuracy and RT). In visual world tasks, phonological competition does not always have an effect on accuracy or RT, and is often only detected with more sensitive measures like eye-tracking or mouse-tracking^35,36^. We did find an interaction between language and competition type in accuracy, where the two conditions that included English competitors (i.e., English within-language and Spanish between-language) were responded to less accurately than the conditions that included Spanish competitors (i.e., Spanish within-language and English between-language). These findings are consistent with the neural results, which indicated that the more proficient and more dominant language exerted a stronger competition effect than the less proficient and less dominant language.

Looking at the functional neuroimaging data, we did not find significant differences in neural activation for within-language competition relative to baseline. There may be two factors contributing to this lack of an effect. First, bilinguals may have more experience resolving within-language competition, as words tend to have more within-language than between-language competitors due to phonological differences across languages. Second, because within-language competition does not increase activation of the non-target language, it may require fewer resources to resolve. There were also no significant differences in brain activation when participants heard English cues and faced competition from Spanish, compared to baseline. This finding could potentially be explained by the source of linguistic competition. Because English is the participants’ more dominant language, facing competition from the less dominant language, Spanish, may be cognitively less taxing and does not require additional neural resources to resolve^37–39^.

Additionally, correlations between bilinguals’ activation of the basal ganglia and Simon inhibition scores indicate that bilinguals with lower cognitive control may compensate by increasing basal ganglia recruitment during more difficult tasks (i.e., between-language competition). This suggests that bilinguals are able to flexibly deploy the necessary neural resources to meet the needs of more complex tasks^40,41^.

In conclusion, bilinguals differ in how they respond to spoken-word competition, depending on whether the source is between- or within-language, or from the more proficient or less proficient language. Specifically, *between*-language competition recruits a larger network of frontal control and basal ganglia regions than *within*-language competition, and competition from the dominant language recruits more neural resources than competition from the less dominant language. Additionally, during more taxing language tasks, bilinguals compensate for lower inhibitory control ability by increasing their activation of the basal ganglia. These findings demonstrate the considerable neural plasticity that enables bilinguals to process speech in spite of linguistic competition from multiple sources.

## Methods

### Participants

Sixteen Spanish-English bilinguals participated. Data from the English Within-Language competition session for 8 participants were previously reported in Marian *et al*.^22^. Participants’ age ranged from 18–32 years, all reported normal or corrected-normal vision and no history of neurological or psychiatric illness, and were right-handed. Bilingual language status was confirmed using the *Language Experience and Proficiency Questionnaire* (LEAP-Q)^42^. Bilinguals were exposed to Spanish from birth and to English by the age of 8 (mean age of English acquisition = 5.00 years, *SD* = 1.69 years); all reported English and Spanish proficiencies of at least a 7 on a 0 (none) to 10 (perfect) scale (mean Spanish proficiency = 8.49, *SD* = 0.92; mean English proficiency = 9.62, *SD* = 0.52; *t*(14) = −4.26, *p* < 0.05). Participants were English-dominant, were more proficient in English, and were living and attending a university in the United States where English was the official language.

Experimental protocols were approved by the institutional review boards at Northwestern University and University of Houston, concordant to the relevant guidelines. Informed consent was obtained from all participants.

### Materials

Four groups of twenty competitor sets were constructed: English within-language (English target, English competitor), English between-language (English target, Spanish competitor), Spanish within-language (Spanish target, Spanish competitor), and Spanish between-language (Spanish target, English competitor). Each English within-language set contained a target word (e.g., *candy*), an English phonological onset competitor (e.g., *candle*), and two filler items that did not share overlap with any other items in the set (stimuli from the English within-language set were identical to those used by Marian *et al*.^22^). The English between-language stimuli contained an English target (e.g., *glue*), a Spanish phonological competitor (e.g., *globo* [balloon]), and two phonologically unrelated fillers. Spanish stimuli were constructed similarly: the Spanish within-language stimuli contained Spanish targets and competitors (e.g., *calabaza* [pumpkin] – *calcetín* [sock]), while the Spanish between-language stimuli contained a Spanish target and English competitor (e.g., *muñeca* [doll] – *moon*).

In addition to the 80 competitor trials (20 from each condition), 80 unrelated trials were created as a baseline. Unrelated trials were formed by modifying a competitor trial, replacing the competitor with an item that did not overlap with the target. Competitor items were re-used as unrelated items for a different target to control for visual familiarity. In total, the materials included 80 unique Targets, 80 unique Competitors, and 160 unique Fillers which were never repeated across languages or conditions (i.e., within vs. between-language trials). Each picture was viewed two times, once in a competitor trial and once in a matched unrelated trial. See Supplementary Information for a full list of stimuli.

Across all conditions, targets and competitors shared an average of 2.49 (*SD* = 0.66) phonemes at onset (English within-language: *M* = 2.40, *SD* = 0.68; English between-language: 2.55, *SD* = 0.69; Spanish within-language: *M* = 2.45, *SD* = 0.69; Spanish between-language: *M* = 2.55, *SD* = 0.60; *F*(3,76) = 0.25, *n*.*s*.). All critical stimuli (targets, competitors, and fillers) were matched on word frequency, orthographic and phonological neighborhood size (*CLEARPOND* ^43^), and concreteness, familiarity, and imageability (English: *MRC Psycholinguistic Database* ^44^; Spanish: *BuscaPalabras* ^45^) across both English and Spanish (all *p*s > 0.05).

Black and white line drawings were obtained for each object from the International Picture Naming Project (IPNP) database^46^ based on high naming consistency in both English and Spanish. Items that were unavailable from IPNP were selected from Google Images and independently normed by 20 Spanish-English bilinguals on Amazon Mechanical Turk (https://www.mturk.com). Naming reliability reached 91.48% (*SD* = 9.88) in English and 84.98% (*SD* = 15.89) in Spanish.

Following the structure of Marian *et al*.^22^, images were placed in the outer four corners of the screen at a visual angle of 13–15°. Target locations were counterbalanced across trials; targets occupied the same quadrant across competitor and matched unrelated trials. Competitors were always located in one of the quadrants adjacent to the target.

The 160 trials were divided into two blocks: an English run (20 English within-language, 20 English between-language, and 40 English unrelated trials), and a Spanish run (20 Spanish within-language, 20 Spanish between-language, and 40 Spanish unrelated trials). Within each block, trials were presented in a pseudo-randomized order (repeated images were separated by at least three trials, and the four quadrants contained an equal number of targets) that was fixed between participants. To control for familiarity effects, half of the participants received the trials in reverse order. Blocks were presented in a counterbalanced order between participants, with half of the participants receiving the English block first and half of the participants receiving the Spanish block first.

### Procedure

Testing occurred across two sessions: the first for cognitive and behavioral assessments and the second for administration of the fMRI task. During the behavioral session, tests of phonological working memory (*Comprehensive Test of Phonological Processing* ^47^), inhibitory control (*Simon Task* ^48^), and language proficiency in both English (*Woodcock Language Proficiency Battery-Revised* ^49^) and Spanish (*Woodcock-Munoz Language Survey-Revised* ^50^) were administered. See Table 3 for participants’ performance summary.

**Table 3:** Cognitive and Linguistic Participant Demographics.

Measure	Mean (SD)
N	16; 5 male
Age	22.71 (3.89)
Years of Formal Education	15.87 (1.30)
English Age of Acquisition (LEAP-Q)	5.00 (1.69)
Spanish Age of Acquisition (LEAP-Q)	Birth
English Proficiency (LEAP-Q)	9.62 (0.52)
Spanish Proficiency (LEAP-Q)	8.49 (0.92)
English Ability (Woodcock)	73.00 (4.40)
Spanish Ability (Woodcock Muñoz)	67.81 (7.91)
Comprehensive Test of Phonological Processing (CTOPP) Digit Span	16.19 (2.74)
Comprehensive Test of Phonological Processing (CTOPP) Non-word Repetition	14.31 (1.40)
Simon Effect (ms)	32.24 (19.18)
Simon Facilitation Score (ms)	18.32 (21.04)
Simon Inhibition Score (ms)	15.92 (14.08)

During the fMRI session, participants were familiarized with the fMRI scanner and all procedures. Participants were given sound-dampening headphones to reduce scanner noise, a squeeze ball to signal the fMRI technician, and a four-button button box. A display of four images was projected onto a mirrored screen, and participants heard auditory instructions to locate one of the four images.

Timing and trial structure was identical to that used by Marian *et al*.^22^. A four-item visual search display was shown for 500 ms before participants heard an auditory target word (spoken by a male Spanish-English bilingual) at 48 Khz, amplitude-normalized. Following the auditory token, the search display remained on the screen for 2500 ms; participants used the button box to indicate target location. Each visual quadrant corresponded to a single button (the bottom left button corresponded to the bottom left quadrant, etc.). Stimuli were presented using E-Prime 2.0 software (Psychology Software Tools, Pittsburgh, PA) in an event-related design with an inter-stimulus interval between 4.5–11.7 seconds.

Following the search task, participants named all competitor pictures in English and Spanish while seated at a computer outside the scanner. Trials in which participants failed to provide a name, or provided an incorrect name were removed from all analyses (6.72% of English trials, 13.6% of Spanish trials).

### Neuroimaging Parameters

Functional neuroimaging data were collected using a 3.0 Tesla head-only Siemens Magnetom Allegra magnetic imager located in Baylor College of Medicine’s Human Neuroimaging Laboratory. Anatomical images were acquired using a high-resolution T_1_-weighted MPRAGE sequence (voxel size = 1.0 × 1.0 × 1.0 mm, TR = 1200 ms, TE = 2.93 ms, reconstructed into 192 slices). Functional images were acquired in 34 axial slices parallel to the AC-PC line with an interleaved descending gradient recalled echo-planar (EPI) imaging sequence (voxel size = 3.4 × 3.4 × 4.0 m, TR = 2700 ms, and TE = 28 ms).

### Data analysis

#### Accuracy and response time

Response time (RT) was measured from the onset of the search display to button response. RTs were analyzed for correct trials only; 2.80% of trials were identified as outliers (longer than the global mean plus 2.5 standard deviations) and were replaced with the threshold value (i.e., *M* + 2.5 *SD*). Accuracy and RT were compared across trial types using logistic (for accuracy) or linear (for RT) mixed effect (LME) regression, using the lme4 package^24^ in the R statistical computing environment^25^. Models included subject and item random effects, and fixed effects of competition (competitor, unrelated), type (between-language, within-language), and presentation language (English, Spanish). Parameter-specific *p* values were estimated by using a normal approximation, treating the *t* value from the model as a *z* value^51^. Follow-up pairwise comparisons were performed using Welch *t*-tests and the Satterthwaite approximation for degrees of freedom, with the Tukey correction for multiple comparisons.

#### Functional neuroimaging

Functional images were analyzed using SPM8 (Wellcome Trust Centre for Neuroimaging, London, UK). Images were realigned for motion correction, resliced, and slice time corrected. Functional images were coregistered to align with the structural image, segmented, and normalized to a standard Montreal Neurological Institute (MNI) template. Data were spatially smoothed using an 8 mm full-width half maximum (FWHM) Gaussian kernal.

In first-level processing, stimulus onsets locked to the auditory stimulus for the four competitor conditions (within-English competitor, between-English competitor, within-Spanish competitor, between-Spanish competitor) were contrasted against their matched unrelated trial onsets (within-English unrelated, between-English unrelated, within-Spanish unrelated, between-Spanish unrelated) in each participant using a General Linear Model (GLM). Motion estimates from preprocessing were entered as covariates of no interest at the first-level to further control for motion artifacts^52^. A 2 (condition: between-language, within-language) × 2 (language: English, Spanish) within-subject ANOVA assessed main effects of condition and language on phonological competition. Secondary analyses in each language were performed using one-way within-subject ANOVAs. Based on cortical activation during linguistic competition^18,53^, analyses were restricted to an ROI including bilateral inferior frontal gyrus, middle frontal gyrus, superior frontal gyrus, inferior parietal lobule, superior parietal lobule, anterior cingulate, and basal ganglia using anatomical definitions in the AAL template^54^. Monte Carlo simulations with AFNI’s ALPHASIM program were performed to correct for multiple comparisons. All comparisons used a voxel-level threshold of *p* < 0.025 and a minimum cluster size of 509 contiguous voxels, for a cluster-level significance of *p* < 0.05.

The relationship between proficiency, inhibitory control, and cortical activation in response to phonological competition was examined using individual effect sizes in task-identified regions of interest (ROIs) following a leave-one-subject-out (LOSO) approach^55^. Sixteen separate LOSO GLMs were performed, each with n = 15. Task-activated ROIs were identified in each model using the same procedure as the full analysis (ROIs identified in less than a third of LOSO GLMs were not analyzed further). For each participant, mean beta weights for within-English, between-English, within-Spanish, and between-Spanish competition effects were calculated in each ROI from the LOSO GLM that excluded that participant, preserving independence of ROI selection and measured task activation. Mean beta weights were correlated with participants’ language proficiency, Simon inhibition scores (RT on incongruent trials minus RT on neutral trials) and overall Simon task RTs. Simon inhibition scores were used instead of the classic Simon effect (i.e., incongruent minus congruent), as the former provides a more targeted measure of interference suppression^56^.

### Data availability

The datasets analyzed are available from the corresponding author on reasonable request.

## Electronic supplementary material

Dataset 1

Dataset 2

Dataset 3

Supplementary Information
