# Supplementary material for: Bilingual Cortical Control of Between- and Within-Language Competition
Source: Sci Rep. 2017 Sep 18;7:11763. doi: 10.1038/s41598-017-12116-w (PMC5603581; doi:10.1038/s41598-017-12116-w)
Supplement: Supplementary file 4 — Supplementary Information [file 41598_2017_12116_MOESM4_ESM.pdf]

Bilingual Cortical Control of Between- and Within-Language Competition

Viorica Marian[a]*, James Bartolotti[a], Sirada Rochanavibhata[a], Kailyn Bradley[c], and Arturo E. Hernandez[b]

[a]Northwestern University, [b]University of Houston, [c]Icahn School of Medicine at Mount Sinai

*Corresponding Author:

Dr. Viorica Marian

Department of Communication Sciences and Disorders

2240 North Campus Drive

Northwestern University

Evanston, IL, 60208-3540

Email: v-marian@northwestern.edu

Phone: (847) 491-2420

Supplementary Information

Supplementary Table S1

Target, Competitor, Unrelated, and Filler Stimuli in the English Block

	Target	Competitor	Unrelated	Filler 1	Filler 2
(A) English Within-Language					
1	**antler** (*cuerno*)	**ant** (*hormiga*)	toe (*dedo*)	raft (*balsa*)	spatula (*pala*)
2	**basket** (*canasta*)	**bat** (*murciélago*)	lightning (*rayo*)	hat (*sombrero*)	diaper (*pañal*)
3	**brain** (*cerebro*)	**bridge** (*puente*)	elevator (*ascensor*)	king (*rey*)	glasses (*lentes*)
4	**candy** (*dulces*)	**candle** (*vela*)	snowman (*mono de nieve*)	hair (*pelo*)	barbecue (*asador*)
5	**drum** (*tambor*)	**dress** (*vestido*)	watering can (*regadera*)	knife (*cuchillo*)	bear (*oso*)
6	**sheep** (*oveja*)	**shield** (*escudo*)	raccoon (*mapache*)	neck (*cuello*)	buckle (*hebilla*)
7	**gun** (*pistola*)	**gutter** (*canalón*)	screw (*tornillo*)	walrus (*morsa*)	tree (*árbol*)
8	**lighter** (*encendedor*)	**lightning** (*rayo*)	cloud (*nube*)	skateboard (*patineta*)	toilet (*inodoro*)
9	**magnet** (*imán*)	**match** (*cerilla*)	witch (*bruja*)	pencil (*lápiz*)	corn (*elote*)
10	**pig** (*cerdo*)	**picture** (*cuadro*)	ant (*hormiga*)	fireplace (*chimenea*)	roof (*techo*)
11	**rattle** (*sonaja*)	**raccoon** (*mapache*)	hummingbird (*colibrí*)	bacon (*tocino*)	pillow (*almohada*)
12	**elbow** (*codo*)	**elevator** (*ascensor*)	bat (*murciélago*)	hanger (*gancho*)	door (*puerta*)
13	**clown** (*payaso*)	**cloud** (*nube*)	match (*cerilla*)	owl (*búho*)	log (*tronco*)
14	**honey** (*miel*)	**hummingbird** (*colibrí*)	shield (*escudo*)	rice (*arroz*)	dog (*perro*)
15	**snail** (*caracol*)	**snowman** (*mono de nieve*)	dress (*vestido*)	whisk (*batir*)	lightbulb (*foco*)
16	**flag** (*bandera*)	**flashlight** (*linterna*)	picture (*cuadro*)	rocking chair (*mecedora*)	staircase (*escalera*)
17	**scarf** (*bufanda*)	**screw** (*tornillo*)	candle (*vela*)	ladder (*escalera*)	hot dog (*perro caliente*)
18	**toad** (*sapo*)	**toe** (*dedo*)	flashlight (*linterna*)	box (*caja*)	ice cream (*helado*)
19	**waterfall** (*cascada*)	**watering can** (*regadera*)	bridge (*puente*)	tape (*cinta*)	moose (*alce*)
20	**wig** (*peluca*)	**witch** (*bruja*)	gutter (*canalón*)	lighthouse (*faro*)	eye (*ojo*)

(B) English Between-Language

	**loop**	magnifying glass	pocket	easel	onion
1	(*rizo*)	**(*lupa*)**	(*bolsillo*)	(*caballete*)	(*cebolla*)
	**engine**	nurse	tie	fish	grapes
2	(*motor*)	**(*enferma*)**	(*corbata*)	(*pez*)	(*uvas*)
	**seagull**	chair	nurse	key	mushroom
3	(*gaviota*)	**(*silla*)**	(*enferma*)	(*llave*)	(*hongo*)
	**desk**	screwdriver	magnifying glass	fireman	chicken
4	(*escritorio*)	**(*desarmador*)**	(*lupa*)	(*bombero*)	(*gallina*)
	**cooler**	spoon	arrow	leg	vase
5	(*hielera*)	**(*cuchara*)**	(*flecha*)	(*pierna*)	(*jarrón*)
	**flame**	arrow	banana	slingshot	cage
6	(*llave*)	**(*flecha*)**	(*pláatno*)	(*tirador*)	(*jaula*)
	**orange**	ear	beach	smoke	drawer
7	(*naranja*)	**(*oreja*)**	(*playa*)	(*humo*)	(*cajón*)
	**platypus**	banana	scissors	thimble	cactus
8	(*ornitorrinco*)	**(*plátano*)**	(*tijeras*)	(*dedal*)	(*nopal*)
	**clock**	nail	shark	watermelon	umbrella
9	(*reloj*)	**(*clavo*)**	(*tiburón*)	(*sandía*)	(*paraguas*)
	**beak**	mustache	screwdriver	vacuum	crackers
10	(*pico*)	**(*bigote*)**	(*desarmador*)	(*aspiradora*)	(*galletas*)
	**teacher**	shark	whistle	bow	rooster
11	(*maestra*)	**(*tiburón*)**	(*silbato*)	(*arco*)	(*gallo*)
	**glue**	balloon	spoon	toothpaste	whip
12	(*pegamento*)	**(*globo*)**	(*cuchara*)	(*dentrífico*)	(*látigo*)
	**marble**	hammer	balloon	lung	rain
13	(*canica*)	**(*martillo*)**	(*globo*)	(*pulmón*)	(*lluvia*)
	**corkscrew**	tie	ear	faucet	wing
14	(*sacacorchos*)	**(*corbata*)**	(*oreja*)	(*grifo*)	(*ala*)
	**plug**	beach	chair	house	jellyfish
15	(*enchufe*)	**(*playa*)**	(*silla*)	(*casa*)	(*medusa*)
	**plow**	feather	hammer	deer	necklace
16	(*arado*)	**(*pluma*)**	(*martillo*)	(*venado*)	(*collar*)
	**cheek**	gum	nail	beer	pipe
17	(*mejilla*)	**(*chicle*)**	(*clavo*)	(*cerveza*)	(*tuberia*)
	**teeth**	scissors	feather	shell	backpack
18	(*dientes*)	**(*tijeras*)**	(*pluma*)	(*concha*)	(*mochilla*)
	**bowl**	pocket	gum	needle	shower
19	(*cuenco*)	**(*bolsillo*)**	(*chicle*)	(*aguja*)	(*regadera*)
	**silverware**	whistle	mustache	yarn	turkey
20	(*objetos de plata*)	**(*silbato*)**	(*bigote*)	(*hilo*)	(*pavo*)

Note. Words in parentheses represent Spanish translations of all items. Bolded words indicate phonologically-overlapping pairs.

Supplementary Table S2

Target, Competitor, Unrelated, and Filler Stimuli in the Spanish Block

	Target	Competitor	Unrelated	Filler 1	Filler 2
(A) Spanish Within-Language					
	whale	cane	fly	skate	nostril
1	**(*ballena*)**	**(*bastón*)**	(*mosca*)	(*patín*)	(*orificio*)
	diver	mailbox	shirt	rock	tongue
2	**(*buzo*)**	**(*buzón*)**	(*camisa*)	(*piedra*)	(*lengue*)
	chain	horse	knee	wheelchair	table
3	**(*cadena*)**	**(*caballo*)**	(*rodilla*)	(*silla de ruedas*)	(*mesa*)
	bell	shirt	hose	wine	slippers
4	**(*campana*)**	**(*camisa*)**	(*manguera*)	(*vino*)	(*zapatillas*)
	pumpkin	sock	mouth	grave	wrench
5	**(*calabaza*)**	**(*calcetín*)**	(*boca*)	(*tumba*)	(*llave*)
	mirror	handcuffs	dove	rope	gate
6	**(*espejo*)**	**(*esposas*)**	(*paloma*)	(*cuerda*)	(*verja*)
	suitcase	hose	octopus	cat	frog
7	**(*maleta*)**	**(*manguera*)**	(*pulpo*)	(*gato*)	(*rana*)
	bone	egg	swing	fox	milk
8	**(*hueso*)**	**(*huevo*)**	(*columpio*)	(*zorro*)	(*leche*)
	umbrella	dove	mailbox	sickle	zipper
9	**(*paraguas*)**	**(*paloma*)**	(*buzón*)	(*hoz*)	(*cremallera*)
	apple	butter	handcuffs	parrot	knight
10	**(*manzana*)**	**(*mantequilla*)**	(*esposas*)	(*loro*)	(*caballero*)
	windmill	fly	cane	shoulder	scale
11	**(*molino*)**	**(*mosca*)**	(*bastón*)	(*hombro*)	(*pesa*)
	duck	shovel	window	fence	cheese
12	**(*pato*)**	**(*palo*)**	(*ventana*)	(*cerco*)	(*queso*)
	drill	heel	shovel	eyebrow	wreath
13	**(*taladro*)**	**(*tacón*)**	(*palo*)	(*ceja*)	(*corono de flores*)
	fan	window	carrot	ball	hand
14	**(*ventilador*)**	**(*ventana*)**	(*zanahoria*)	(*pelota*)	(*mano*)
	puzzle	knee	horse	arm	sunflower
15	**(*rompecabezas*)**	**(*rodilla*)**	(*caballo*)	(*brazo*)	(*girasol*)
	cow	glass	heel	wolf	mermaid
16	**(*vaca*)**	**(*vaso*)**	(*tacón*)	(*lobo*)	(*sirena*)
	shoe	carrot	egg	pan	butterfly
17	**(*zapato*)**	**(*zanahoria*)**	(*huevo*)	(*sartén*)	(*mariposa*)
	purse	mouth	sock	ghost	toys
18	**(*bolsa*)**	**(boca)**	(*calcetín*)	(*fantasma*)	(*juguetes*)
	rocket	swing	glass	chest	acorn
19	**(*cohete*)**	**(*columpio*)**	(*vaso*)	(*pecho*)	(*bellota*)
	thumb	octopus	butter	celery	lock
20	**(*pulgar*)**	**(*pulpo*)**	(*mantequilla*)	(*apio*)	(*canado*)

(B) Spanish Between-Language

	seal	**folder**	leaf	vest	mop
1	**(*foco*)**	(*carpeta*)	(*hoja*)	(*chaleco*)	(*trapeador*)
	beaver	**cast**	moon	pot	wheel
2	**(*castor*)**	(*yeso*)	(*luna*)	(*olla*)	(*rueda*)
	thread	**eel**	folder	cymbals	mouse
3	**(*hilo*)**	(*anguila*)	(*carpeta*)	(*platillo*)	(*ratón*)
	nut	**tweezers**	goat	helmet	skirt
4	**(*tuerca*)**	(*pinzas*)	(*chiva*)	(*casco*)	(*falda*)
	doll	**moon**	cast	sword	jar
5	**(*muñeca*)**	(*luna*)	(*yeso*)	(*espada*)	(*frasco*)
	bride	**notebook**	eel	traffic light	puppet
6	**(*novia*)**	(*cuaderno*)	(*anguila*)	(*semáforo*)	(*marioneta*)
	hut	**choke**	tail	stapler	brick
7	**(*choza*)**	(*ahogar*)	(*cola*)	(*grapadora*)	(*ladrillo*)
	raindrop	**goat**	tweezers	wallet	maze
8	**(*gota*)**	(*chiva*)	(*pinzas*)	(*cartera*)	(*laberinto*)
	claw	**garlic**	plunger	ring	stool
9	**(*garra*)**	(*ajo*)	(*desatascador*)	(*anillo*)	(*banco*)
	weather	**cleaner**	choke	donkey	spider
10	**(*clima*)**	(*limpiador*)	(*ahogar*)	(*burro*)	(*araño*)
	web	**tail**	soap	ostrich	present
11	**(*telaraña*)**	(*cola*)	(*jabón*)	(*avestruz*)	(*regalo*)
	envelope	**soap**	meat	paintbrush	fin
12	**(*sobre*)**	(*jabón*)	(*carne*)	(*brocha*)	(*aleta*)
	hinge	**bees**	notebook	kite	funnel
13	**(*bisagra*)**	(*abejas*)	(*cuaderno*)	(*papalote*)	(*embudo*)
	iron	**plunger**	bees	walnut	stroller
14	**(*plancha*)**	(*desatascador*)	(*abejas*)	(*nuez*)	(*carriola*)
	rabbit	**comb**	garlic	waiter	tire
15	**(*conejo*)**	(*piene*)	(*ajo*)	(*mesero*)	(*llanta*)
	blender	**leak**	tent	squirrel	referee
16	**(*licuadora*)**	(*gotera*)	(*carpa*)	(*ardilla*)	(*arbitro*)
	half	**meat**	leak	bull	ashtray
17	**(*mitad*)**	(*carne*)	(*gotera*)	(*torro*)	(*cenicero*)
	book	**leaf**	comb	skunk	typewriter
18	**(*libro*)**	(*hoja*)	(*peine*)	(*zorrillo*)	(*máquina de escribir*)
	church	**eagle**	cleaner	paper	strawberry
19	**(*iglesia*)**	(*águila*)	(*limpiador*)	(*hojas*)	(*fresa*)
	fork	**tent**	eagle	net	bread
20	**(*tenedor*)**	(*carpa*)	(*águila*)	(*red*)	(*pan*)

Note. Words in parentheses represent Spanish translations of all items. Bolded words indicate phonologically-overlapping pairs.
